# The MASCC COG-IMPACT: An unmet needs assessment for cancer-related cognitive impairment impact developed by the Multinational Association of Supportive Care in Cancer

**DOI:** 10.1007/s00520-025-09149-7

**Published:** 2025-01-24

**Authors:** Darren Haywood, Alexandre Chan, Raymond J. Chan, Frank D. Baughman, Evan Dauer, Haryana M. Dhillon, Ashley M. Henneghan, Blake J. Lawrence, Maryam B. Lustberg, Moira O’Connor, Janette L. Vardy, Susan L. Rossell, Nicolas H. Hart

**Affiliations:** 1 Human Performance Research Centre, INSIGHT Research Institute, Faculty of Health, University of Technology Sydney (UTS), Moore Park, Sydney, NSW 2030 Australia; 2 Department of Mental Health, St Vincent’s Hospital Melbourne, Fitzroy, VIC Australia; 3 Department of Psychiatry, Faculty of Medicine, Dentistry and Health Sciences, University of Melbourne, Melbourne, VIC Australia; 4 School of Population Health, Faculty of Health Sciences, Curtin University, Bentley, WA Australia; 5 School of Pharmacy and Pharmaceutical Sciences, University of California, Irvine, USA; 6 Caring Futures Institute, College of Nursing and Health Sciences, Flinders University, Adelaide, SA Australia; 7 Psycho-Oncology Cooperative Research Group, School of Psychology, Faculty of Science, University of Sydney, Sydney, Australia; 8 School of Nursing, University of Texas at Austin, Austin, TX USA; 9 Department of Oncology, Dell Medical School, The University of Texas at Austin, Austin, TX USA; 10 Yale University School of Medicine, New Haven, CT USA; 11 Sydney Medical School, Faculty of Medicine and Health, The University of Sydney, Sydney, Australia; 12 Centre for Mental Health and Brain Sciences, Swinburne University of Technology, Hawthorn, VIC Australia; 13 Cancer and Palliative Care Outcomes Centre, Faculty of Health, Queensland University of Technology (QUT), Brisbane, QLD Australia; 14 Exercise Medicine Research Institute, School of Medical and Health Science, Edith Cowan University, Joondalup, WA Australia; 15 Institute for Health Research, University of Notre Dame Australia, Fremantle, WA Australia

**Keywords:** Cancer-related cognitive impairment, Needs, Unmet needs, Assessment, Cancer

## Abstract

**Purpose:**

Cancer-related cognitive impairment (CRCI) can have a profound impact on the lives of cancer survivors. A multitude of subjective and objective assessment tools exist to assess the presence and severity of CRCI. However, no purpose-built tool exists to assess the unmet needs of cancer survivors directly relating to CRCI. This paper details the development and initial validation of the *Multinational Association of Supportive Care in Cancer - Unmet Needs Assessment of Cancer-Related Cognitive Impairment Impact (the MASCC COG-IMPACT).*

**Methods:**

A multistep mixed-methods measurement development and validation approach was taken with a strong emphasis on co-design. Qualitative interviews were conducted with cancer survivors (*n* = 32) and oncology health professionals (*n* = 19), followed by a modified Delphi survey with oncology health professionals (*n* = 29). Cognitive interviews with cancer survivors (*n* = 22) over two rounds were then conducted to finalise the penultimate version of the unmet needs assessment tool for CRCI. Four-hundred and ninety-one (*n* = 491) cancer survivors then completed the MASCC COG-IMPACT and other established measures to inform structural, reliability, validity, acceptability, appropriateness, and feasibility analyses.

**Results:**

The final MASCC COG-IMPACT is a 55-item and eight subscale tool including two indices: “difficulties” and “unmet needs”. The MASCC COG-IMPACT was found to have strong structural validity, convergent validity, discriminant validity, internal consistency, and test–retest reliability. The MASCC COG-IMPACT was also found to be highly acceptable, appropriate, and feasible.

**Conclusion:**

The MASCC COG-IMPACT may facilitate optimal care and referral in line with a cancer survivor’s CRCI-related difficulties and unmet needs. The MASCC COG-IMPACT may also be used to explore factors and contributors to CRCI-related difficulties and unmet needs. Overall, the MASCC COG-IMPACT is a highly reliable and valid tool for the assessment of CRCI-related difficulties and unmet needs in both clinical and research settings. The MASCC COG-IMPACT and supporting materials can be accessed on the MASCC webpage or via the MASCC COG-IMPACT Open Science Framework webpage (https://osf.io/5zc3a/).

**Supplementary Information:**

The online version contains supplementary material available at 10.1007/s00520-025-09149-7.

## Introduction

Most cancer survivors (up to 75%) experience negative changes to their cognitive functioning, impacting their ability to think quickly and clearly, reason, form judgements, and make decisions. These cognitive changes, aptly named cancer-related cognitive impairments (CRCI), are thought to occur due to cancer, cancer treatment(s), and psychosocial distress [1–3]; and can persist decades after treatment completion [1, 2, 4–6]. While prior research examining CRCI has historically focused on chemotherapy and breast cancer, these negative cognitive changes have been observed across different treatments, cancer types, ages, and cancer stages [1, 7]. Indeed, CRCI has a profound impact on cancer survivors’ lives across multiple domains, including their ability to perform daily activities, occupational functioning, social functioning, relationship functioning, and psychological well-being [5, 8–10]. The significant effects of CRCI resulted in cognitive functioning being included as a rehabilitation target in the World Health Organisation’s Package of Interventions for Rehabilitation (Cancer) [11].

A multitude of treatments and supportive care approaches have been developed to minimise the severity or impact of CRCI, including pharmacological treatments, cognitive remediation and training, psychological therapy, physical activity and exercise interventions, mind–body interventions, occupational therapy, support groups, and family or carer supportive care programs [2, 4, 6, 12–17]. While treatment and supportive care approaches for CRCI are still developing, many of these approaches show promise in reducing CRCI or its impacts [18, 19]. A range of tools have also been developed to assess the presence and severity of CRCI, including objective neurocognitive testing, and computerised or paper-based tasks, as well as subjective self-report measures of cognitive functioning, with some of these measures including performance or rating “thresholds” that can be used to facilitate determining the presence of CRCI [1, 6, 20, 21]. While a significant body of literature exists to assess CRCI presence and severity, there is currently no purpose-built assessment tool to measure the unmet supportive care and informational needs of cancer survivors facing the challenges specifically associated with CRCI [5, 10].

Health professionals are aware of CRCI and its potential impacts on cancer survivors, however commonly report being under-resourced to assess the unmet needs of an individual with CRCI due to the lack of a purpose-built tool [5, 10]. Health professionals also report this affects their ability to provide optimal care and referral for cancer survivors with CRCI to address these unmet needs [5, 10]. While there are existing unmet needs assessment tools for cancer survivors, these do not provide information specific to the impacts of CRCI, and their scope is typically much broader, encompassing domains unrelated to CRCI (i.e., sexual functioning, numbness, hair loss, etc.) [22, 23]. The remaining available assessment tools conversely examine cognitive impacts for cancer survivors on single-issue and narrow domains such as work [24]. The issues of excessive breadth or specificity deter healthcare professionals from using the existing assessment tools to understand CRCI-related unmet needs [5, 10].

Optimal care for CRCI should be highly individualised and depends on cancer survivors' specific contexts and requirements [6, 8]. For example, two people with the same or similar CRCI characteristics (i.e., cognitive state and change profile) may have very different unmet needs, and thus supportive care requirements, due to their unique life circumstances (i.e., employment, responsibilities, treatment history, available support networks, etc.). To facilitate the delivery of optimal care, health professionals require a purpose-built unmet needs assessment for CRCI for use with existing objective and subjective cognitive functioning assessments that facilitate identifying the presence of CRCI (i.e., together, these tools could identify the presence, severity, and associated unmet needs of CRCI for each person). Accordingly, a purpose-built unmet needs assessment for CRCI should facilitate appropriate referral and support service provision for cancer survivors, guide further assessment, encourage and facilitate clinical or healthcare discussions, and be used to assess intervention efficacy over time.

In this paper, we therefore detail the development and initial validation of the first purpose-built unmet needs assessment for CRCI: *the Multinational Association for Supportive Care in Cancer - Unmet Needs Assessment of Cancer-Related Cognitive Impairment Impact (MASCC COG-IMPACT)*. The MASCC COG-IMPACT is an official tool of MASCC.

## Methods and materials

A detailed description of the materials and methods used to develop and validate this purpose-built unmet needs assessment tool for CRCI is provided in the published protocol [9]. Herein, a high-level overview is provided.

### Design

A multi-stage mixe-methods design was adopted and was informed by the approaches taken to develop other established oncology and haematology unmet needs assessment tools [e.g., 22, 25, 26]. However, this project incorporated additional steps to elevate its suitability and utility, with a greater focus on co-design that involved cancer survivors and oncology health professionals and experts. Ethics approval was obtained from St. Vincent’s Hospital Melbourne Human Research Ethics Committee prior to data collection (PID05582).

### Procedure

This project utilised an eight-step procedure grounded in the lived experience of cancer survivors and oncology healthcare professionals, as summarised below:

- *Step 1*. Thirty-two (*n* = 32) semi-structured interviews were conducted with cancer survivors who reported to be experiencing CRCI symptoms.
- *Step 2.* Nineteen (*n* = 19) semi-structured interviews were conducted with a range of health professionals who work with people affected by cancer and CRCI.
- *Step 3*. Initial assessment domains and an extended item bank were developed, which were informed by the interviews within Steps #1 and #2.
- *Step 4*. A modified single-round Delphi survey [27], involving twenty-nine (*n* = 29) oncology health professionals and experts who directly work with people experiencing CRCI, on the extended item bank (183-items) was conducted. Health professionals and experts endorsed their five top items for each of the 11 domains/subdomains developed in step #3.
- *Step 5*. Twenty-two (*n* = 22) multi-round cognitive interviews [28] were performed with cancer survivors experiencing CRCI, over Zoom, telephone, or through text-based asynchronous methods depending on the preferences of the participant. First, a Reparative Approach was used, focusing on improving items and instructions [28]. Next, a Descriptive Approach was used, focusing on participant interpretations thereby ensuring the items are being interpreted as intended and measuring the intended domains [28].
- *Step 6*. Refinement of the draft MASCC COG-IMPACT assessment was completed based on the feedback received from the cognitive interviewing process. Steps #5 and #6 were repeated twice to iteratively improve the measure.
- *Step 7.* The MASCC COG-IMPACT assessment, as well as other validated measures (see measures section) was administered to four-hundred and ninety-one (*n* = 491) cancer survivors reporting to be experiencing CRCI worldwide. The MASCC COG-IMPACT was then re-administered 2 weeks following the first completion to enable the assessment of test re-test reliability.
- *Step 8.* The structure, reliability, validity, acceptability, appropriateness, and feasibility of the MASCC COG-IMPACT were then assessed (see analysis section).

### Participants

#### Cancer survivors

Cancer survivor participants were 18 years or older; with a prior diagnosis of cancer (any type); had received and completed cancer treatment with curative intent (any treatment); had no current evidence of disease; personally perceived to experience CRCI; and were fluent in English (reading and speaking). The only exclusion criterion was the diagnosis of another neurocognitive or neurological disorder.

#### Oncology health professionals and experts

Oncology health professional and expert participants were 18 years or older; from any health professional discipline; are working, or have worked directly with cancer survivors who have experienced CRCI; and were fluent in English (reading and speaking). The only exclusion criterion was oncology health professionals and experts who had not worked with cancer survivors experiencing CRCI.

### Recruitment

Participants involved in Steps #1, #2, #4, and #5 were recruited using convenience and snowball sampling from the wider community (via social media posts), and via oncology organisations, research groups, and societies. Participants involved in Step #7 were recruited via the research recruitment platform Prolific [29]. Prolific is a highly respected participant sourcing platform that is valid and reliable, and has been extensively used worldwide, including widely for measurement development [30–34].

### Measures

The measures used in Steps #1 and #2 (i.e., interview schedule, demographic, and clinical) are described in the previously published qualitative research [5] and the protocol [9]. The modified-Delphi utilised an online survey checklist via Qualtrics with 183-items (see the protocol for full details [9]). Cognitive interviewing (Step #5) included questions informed by previous oncology and haematology measurement development interview approaches [35] such as “how do you think these instructions could be made clearer” (reparative approach), and “In your own words, what do you think this group of questions is asking about” (descriptive approach). The self-report survey (Step #7) included (1) demographic and clinical questions, (2) the MASCC COG-IMPACT, (3) the Depression Anxiety, and Stress Scale-21 (DASS-21) [36], (4) Cancer Survivors Unmet Needs Scale (CaSUN) [26], (5) Assessment of Quality of Life Scale (AQoL) 6D [37], (6) the Cognitive Symptom Checklist-Work (CSC-W) [24], (7) the Patient-Reported Outcomes Measurement Information System-Cognitive Function Scale 8a (PROMIS-COG) [38], and (8) the adapted Acceptability of Intervention Measure (AIM), Intervention Appropriateness Measure (IAM), and Feasibility of Intervention Measure (FIM) [39]. The survey was administered online via Qualtrics. See the protocol for full detail [9].

### Analysis

#### Qualitative

Qualitative analysis for Steps #1, #2, and #5 utilised reflexive thematic analysis aided by the Nvivo software. Analysis followed the guidelines outlined by Braun and Clarke [36, 37] (see the protocol [9] and qualitative phase published separately [5]).

#### Quantitative

##### Modified-Delphi

Frequencies and descriptive analysis, such as number of votes, and percentage of votes were calculated to determine the most commonly endorsed items for each subdomain from the item bank.

##### Structural analysis

An exploratory Principal Components Analysis (PCA), using an oblique (oblimin) rotation was conducted in accordance with the specifications of Allan et al. [40]. This is standard for measurement development of this type and is highly utilised in oncology measurement development and psychometric assessment, including within CRCI [e.g., 24, 41, 42]. Suitability of the data for PCA was assessed using the exploration of item bivariate correlations, the determinant value, the Kaiser–Meyer–Olkin Measure of Sampling Adequacy (KMO), the item-level Measure of Sampling Adequacy, and Bartlett’s Test of Sphericity. Final structure and item inclusion were chosen based on eigenvalues of > 1, cumulative variance explained, item factor loadings, Cronbach’s alpha if an item was retained or removed, and theoretical and clinical interpretation. As per standard practice [40], and previous unmet needs assessment development in cancer [e.g., 22], factor loadings of > 0.3 were used as a cut-off to facilitate item retention or removal decisions.

##### Reliability

Internal consistency was assessed using Cronbach’s Alpha and interpreted based on established thresholds of “excellent” = ≥ 0.90, “very good” = 0.90-0.81, “good” = 0.80-0.71, “moderate” = 0.70-0.61, and “poor” = < 0.60. Test–retest reliability was assessed using intra-class correlation (ICC) analysis between the initial administration and the 2-week re-rest administration of the COG-IMPACT. Test–retest reliability was interpreted based on the following established thresholds “excellent” > 0.75, “good” = 0.75–0.61, and “fair” = 0.60–40 [43].

##### Convergent validity

Convergent validity was assessed through bivariate correlations between the MASCC COG-IMPACT subscales and established assessments. Specifically, due to the known interrelation between health and wellbeing metrics and unmet need domains, it was expected each MASCC COG-IMPACT subscale would be significantly positively associated with each of the PROMIS-COG, CSC-W, CaSUN, DASS-21, and AQoL subscales and applicable overall scores.

##### Discriminant validity

Discriminant validity was assessed through bivariate correlations between the MASCC COG-IMPACT subscales and established measures. It was expected correlations between MASCC COG-IMPACT subscales and established measures would discriminate by domain theoretically-related associations exceeding those of lesser theoretically-related domains. It was further expected that correlations between the MASCC COG-IMPACT subscales and the AQoL-Senses subscale would not exceed a ‘small’ positive correlation (i.e., all correlation would be *r *< 0.4; [44]), due to the minimal theoretical association between CRCI-related unmet needs and sensory difficulties.

##### Acceptability, appropriateness, and feasibility

Acceptability, appropriateness, and feasibility were analysed using descriptive and frequency statistics of the Weiner et al. [39] measures. Furthermore, in line with previous unmet needs measurement developments [e.g., 25], the appropriateness of the individual items was also assessed using item-response frequencies. As per previous measurement development efforts, an item was determined as appropriate if it was endorsed as a “difficulty” for > 10% of participants [25].

## Results

Results of Steps #1 and #2 have been published separately [5]. In summary, the initial qualitative phase resulted in the development of six themes (1. Executing regular activities, 2. Relational Difficulties, 3. Occupational Functioning, 4. Psychosocial Distress, 5. Social functioning and 6. Informational Needs), and 10 sub-themes (1.1 Difficulties in Daily Tasks, 1.2 Difficulty Engaging in Valued Activities, 2.1 Impacts on Intimate Relationships, 2.2 Difficulty Parenting, 3.1 Decreased Work Capacity, 3.2 Difficulty Returning to Work, 4.1 Loss of Confidence, 4.2 Frustration and Distress, 5.1 Difficulty in Conversation, 5.2 Social Avoidance).

### Modified Delphi

Twenty-nine (*n* = 29) oncology health professionals and experts who self-identified as directly having previously or currently working with people experiencing CRCI completed the single-round modified Delphi item-endorsement survey. Detailed demographic information of the participants is provided in the Online Resource Supplementary Table 1. Item endorsement counts for all 183-items are provided in the Online Resource Supplementary Table 2. In brief, participants were from 13 different countries (most commonly Australia (34.5%) and the USA (24.1%)), 10 different disciplines/roles, (most commonly Academic Researcher (44.8%), Nurse (27.6%), and Physiotherapist or Occupational Therapist (20.7%)), the majority were female (82.8%), and the mean number of years working in oncology at 17.1 (SD = 10.5).

Four of the 11 domains and subdomains included two items that tied as the fifth most commonly endorsed item. Ties were resolved by the research team based on the face and content validity of the items for each domain/subdomain. The modified Delphi process resulted in 55 draft items in total to be included in the provisional MASCC COG-IMPACT to be presented to cancer survivors in the cognitive interviews.

### Cognitive interviews

Twenty-two (*n* = 22) cancer survivors participated in a total of 29 cognitive interviews over two rounds. Demographic information of the participants across the two rounds are provided in the Online Resource Supplementary Tables 3 and 4. In brief, participants had a mean age of 56.8 (SD = 8.8) and were mostly female (77.3%). The most common primary cancer types were breast (63.6%) and lymphoma (27.3%), and the most commonly received treatments were chemotherapy (68.2%), surgery (54.5%), and radiation (40.9%). The cognitive interviewing process resulted in revisions to measure instructions and response options, as well as revisions to item wording and the replacement of one draft item with an alternative item from the extended item bank due to cancer survivor perspectives regarding the content validity of each domain.

The response format preferred was an indication of the presence or absence of listed CRCI-related “difficulty” (each item) in a “no”/ “yes” format, and if “yes” was selected, an indication about the level of unmet supportive care need for the difficulty would be required. Response options for the level of unmet supportive care need were in the following Likert Type format “I do not need any additional support,” “my need for support is satisfied,” “I have a low need for additional support,” “I have a moderate need for additional support,” and “I have a high need for additional support.” This process resulted in two indices for the MASCC COG-IMPACT: (1) “Difficulties” and (2) “Unmet Needs.”

### Item scoring

The scoring approach was developed, based on the results of the cognitive interviews and oncology health professional preferences, and is presented in Table 1. This scoring approach was used for all of the following analyses.

**Table 1:** Item scoring

Difficulties Item Scoring
No = 0
Yes = 1
Unmet Needs Item Scoring
‘No’ to ‘Difficulty’ response option = 0
I do not need any additional support = 0
My need for support is satisfied = 0
I have a low need for additional support = 1
I have a moderate need for additional support = 2
I have a high need for additional support = 3

### Structural analysis

Four-hundred and ninety-four cancer survivors completed the MASCC COG-IMPACT and validation measures, however three participants were removed for failing ≥ 50% of the included attention checks (i.e., two or more out of four) leaving a final sample of 491 participants. The demographic and clinical information for the participants is summarised in Table 2. Demographic and clinical information is provided in greater detail in the Online Resource Supplementary Table 5. In brief, participants were from 23 different countries and represented 18 different listed primary cancer types. The most common countries of residence were the USA (35.6%), South Africa (24.2%), and the UK (23.0%). The most common primary cancers were breast (33.4%), lymphoma (7.9%), and bowel/colorectal (6.5%). The most common treatments received were chemotherapy (68.4%), radiation (51.1%), and hormone therapy (27.1%). This data was used for the structural analysis.

**Table 2:** Sample characteristics of cancer survivors completing the validation survey

Characteristic	Mean (SD)/Range/Count
Age (years)	M = 44.39 years (SD = 15.02, Min = 18, Max = 81)
Sex at Birth	
Male	132 (26.9%)
Female	359 (73.1%)
Ethnicity	
Caucasian	290 (59.1%)
African/African American	152 (31.0%)
Asian	17 (3.5%)
Hispanic or Latino	12 (2.4%)
Native American / American Indian	3 (0.6%)
Other	17 (3.5%)
Primary Cancer Type(s)	
Breast	169 (34.4%)
Prostate	22 (4.5%)
Bowel/Colorectal	32 (6.5%)
Melanoma	18 (3.7%)
Lung	26 (5.3%)
Lymphoma	39 (7.9%)
Leukaemia	28 (5.7%)
Brain	14 (2.9%)
Pancreatic	3 (0.6%)
Myeloma	3 (0.6%)
Cervical	21 (4.3%)
Thyroid	30 (6.1%)
Testicular	17 (3.5%)
Uterine	14 (2.9%)
Ovarian	19 (3.9%)
Sarcoma	5 (1.0%)
Kidney	5 (1.0%)
Bladder	4 (0.8%)
Other	22 (4.5%)
Five Most Common Country of Residence	
USA	175 (35.6%)
South Africa	119 (24.2%)
UK	113 (23.0%)
Australia	15 (3.1%)
Poland	10 (2.0%)
Treatments Received	
Chemotherapy	336 (68.4%)
Radiation	256 (52.1%)
Hormone treatment	133 (27.1%)
Targeted Therapies	66 (13.4%)
Surgery	110 (22.4%)
Immunotherapy	78 (15.9%)
Other	11 (2.2%)

Rounding my result in percentages not equalling 100%

The 55 MASCC COG-IMPACT items were deemed highly suitable for factor analysis as the Determinant was > 0.00001, KMO = 0.964, Bartlett’s Test of Sphericity *p* < 0.001, and all item MSA > 0.9. The PCA using an oblique (oblimin) rotation extracted eight factors with eigenvalues > 1 explaining a cumulative 67.97% of variance. The rotated eight-factor solution of the MASCC COG-IMPACT is provided in Table 3.

**Table 3:** Rotated eight-factor solution of the MASCC COG-IMPACT

*Because of my CRCI…*	Factor							
	1	2	3	4	5	6	7	8
I feel that I have lost my self-confidence	**0.856**							
I struggle to feel confident in my ability to cope with life’s challenges	**0.766**							
I feel unsure of myself and my abilities	**0.692**							
I struggle with a sense of loss for who I once was	**0.638**							
I am feeling overwhelmed	**0.612**							
I get frustrated as I am not good at things I used to be	**0.597**							
I feel I can’t trust myself	**0.596**							
I feel like a different person compared to who I was before cancer	**0.531**							
I often get frustrated when I cannot remember something	**0.521**							
I am struggling with anxiety	**0.428**			0.307				0.315
I have difficulty remembering what I intend to do in my day		**0.763**						
I often forget things I need in life (e.g., pin numbers, passwords, etc.)		**0.762**						
I forget things I need (e.g., keys, wallet, etc.)		**0.734**						
I often forget instructions health professionals have given me		**0.503**				0.314		
I have stopped doing things I enjoy that require too much mental effort		**0.500**					0.322	
I have to make more of an effort to perform my daily tasks		**0.456**						
I have trouble remembering important events for my partner/family members/friends		**0.360**			0.307			
I need specific strategies to feel more comfortable returning to work/volunteering/school			**0.760**					
I do not know what my working/volunteering/schooling capacity is			**0.760**					
I struggle with feeling ready to work/volunteer/school			**0.730**					
I feel I cannot work/volunteer/school at my previous capacity			**0.700**					
I need to understand what changes are required to return to work/volunteering/school			**0.640**					
I need some accommodations at work/volunteering/school to better cope			**0.567**					
I have difficulty understanding complex ideas, concepts or processes at work/volunteering/school			**0.450**				−0.318	
I am concerned about letting others down at work/volunteering/school			**0.441**					
Others perceive me differently at work/volunteering/school			**0.436**					
I have difficulty with certain tasks at work/volunteering/school			**0.395**				−0.374	
I need information about what to expect about CRCI				**0.754**				
I need help finding or accessing resources that I can give to others				**0.730**				
I am not sure if my experience is normal				**0.647**				
I need to be informed about what things I can do to help myself manage or improve				**0.643**				
I sometimes fear that I am losing my mind, going crazy, or that I am experiencing early signs of dementia				**0.577**				
There is a change in the dynamic of my relationship(s) with my partner/family members/friends					**0.750**			
Changes to my emotional state have impacted my relationships with my partner/family members/friends					**0.712**			
I have trouble managing the pressures of being a partner/family member/friend					**0.631**			
My partner/family members/friends have difficulty understanding some things I do or struggle with					**0.613**			
I have trouble keeping up with the requirements of being a partner/family member/friend					**0.563**			
I feel I am a burden to my partner/family members/friends					**0.554**			
I struggle with feelings of guilt because of the impact on my partner/family members/friends					**0.538**			
My partner/family members/friends has taken on more at home					**0.463**			
I am concerned that a partner/family member/friend will joke about my forgetfulness					**0.451**			
I cannot find words easily						**0.748**		
I lose my train of thought in a conversation						**0.739**		
I cannot remember details I should remember in a conversation						**0.696**		
I often have to ask people to repeat themselves in conversation						**0.428**		0.368
I am having trouble engaging with things I find meaningful							**0.502**	
I have trouble enjoying things I used to enjoy		0.302					**0.456**	
I have stopped or reduced doing the things I enjoy							**0.450**	
I have trouble engaging in hobbies I enjoy		0.369					**0.373**	
I feel anxious in social situations								**0.855**
I tend to make excuses to get out of social interactions								**0.814**
I am isolating myself from others								**0.805**
I have withdrawn from social activities I used to enjoy								**0.675**
I am drained of energy after social interactions								**0.634**
I tend to be more quiet than usual in group conversations								**0.519**

Extraction Method: Principal Component Analysis. Rotation Method: Oblimin with Kaiser Normalization. Rotation converged in 23 iterations. Loadings < 0.3 are hidden. Bold = Belonging to that factor

Overall, the factors that emerged strongly reflected the themes developed in the initial qualitative phase (Step #1—#3; [5]), with five of the eight factors reflecting the overarching themes and three of the eight factors reflecting subthemes. The eight factors (in order of variance explained) were (1) Psychological Challenges (10 items), (2) Executing Regular Activities (7 items), (3) Occupational/Vocational Functioning (10 items), (4) Informational Needs (5 items), (5) Relational Difficulties (9 items), (6) Verbal Communication Challenges (4 items), (7) Finding Meaning and Enjoyment in Activities (4 items), and (8) Social Functioning and Withdrawal (6 items).

The bolded factor loads presented in Table 3 represent the items belonging to each factor. All of the 55 items loaded on at least one factor at > 0.3, thus all items were retained. Most items had a primary loading on a single factor, however some items cross-loaded on two or more factors. All of the items with cross-loadings had a stronger loading on their expected factor (in line with the qualitative themes) with the expectation of a single item; “I have trouble remembering important events for my partner/family members/friends,” which was expected to have a primary loading on “Relational Difficulties,” loading stronger on “Executing Regular Activities” (0.360) than “Relational Difficulties” (0.307). Upon additional consultation with health professionals and cancer survivors, the item was retained in the “Executing Regular Activities” factor due to factor loading and the subject of the item closely thematically corresponding to the factor.

### Reliability

Internal consistency analysis of each factor, assessed by Cronbach’s Alpha, revealed all eight factors across the “difficulties” indices to have “good” or “very good” internal consistency (ranging from 0.742 to 0.886; see Table 4), and all eight factors across the “unmet needs” indices to have “very good” or “excellent” internal consistency (ranging from 0.879 to 0.943; see Table 4). Furthermore, 119 participants completed the re-test of the COG-IMPACT. The ICC between the two administrations of the MASCC COG-IMPACT found all factors across both “difficulties” (ranging from 0.724 to 0.869; see Table 4) and “unmet needs” (ranging from 0.691 to 0.856; see Table 4) indices to have “good” or “excellent” test–retest reliability.

**Table 4:** Reliability assessment

Factor		Cronbach’s Alpha	Test Re-Test ICC^a^ (*n* = 119)
Psychological Challenges			
	*Difficulties*	0.875 (very good)	0.855** (excellent)
	*Unmet Needs*	0.943 (excellent)	0.828** (excellent)
Executing Regular Activities			
	*Difficulties*	0.745 (good)	0.822** (excellent)
	*Unmet Needs*	0.870 (very good)	0.814** (excellent)
Occupational/Vocational Functioning			
	*Difficulties*	0.869 (very good)	0.858** (excellent)
	*Unmet Needs*	0.920 (excellent)	0.856** (excellent)
Informational Needs			
	*Difficulties*	0.781 (good)	0.724** (good)
	*Unmet Needs*	0.886 (very good)	0.691** (good)
Relational Difficulties			
	*Difficulties*	0.857 (very good)	0.869** (excellent)
	*Unmet Needs*	0.921 (excellent)	0.820** (excellent)
Verbal Communication Challenges			
	*Difficulties*	0.742 (good)	0.819** (excellent)
	*Unmet Needs*	0.879 (very good)	0.770** (excellent)
Finding Meaning and Enjoyment in Activities			
	*Difficulties*	0.847 (very good)	0.748** (good)
	*Unmet Needs*	0.905 (excellent)	0.727** (good)
Social Functioning and Withdrawal			
	*Difficulties*	0.870 (very good)	0.813** (excellent)
	*Unmet Needs*	0.928 (excellent)	0.777** (excellent)

**Correlation is significant at the 0.01 level. ICC = Intra-Class Correlation Coefficient. ^a^ Type A intraclass correlation coefficients using an absolute agreement definition. This estimate is computed assuming the interaction effect is absent, because it is not estimable otherwise

### Convergent validity

Bivariate correlations between overall and subscale scores of all validation measures and MASCC COG-IMPACT are provided in Table 5. As expected, all of the associations between all MASCC COG-IMPACT subscale scores, for both the “difficulties” and “unmet needs” indices, and the validation measures subscale and overall scores were significant (*p* < 0.001). Furthermore, as expected, all associations between the MASCC COG-IMPACT and validation measures were positive, meaning that greater severity of CRCI-related difficulties and unmet needs were related to poorer subjective cognition, greater work-related cognitive symptoms, greater general unmet needs, lower quality of life, and greater depression, anxiety, and stress. Overall, the MASCC COG-IMPACT was found to have strong convergent validity.

**Table 5:** Bivariate correlations between the MASCC COG-IMPACT and validation measures

	ERA DIFF	ERA UN	FME DIFF	FME UN	RD DIFF	RD UN	OVF DIFF	OVF UN	PC DIFF	PC UN	VCD DIFF	VCD UN	SFW DIFF	SFW UN	IN DIFF	IN UN
PROMIS-COG	0.658**	0.389**	0.483**	0.396**	0.573**	0.444**	0.576**	0.463**	0.661**	0.551**	0.639**	0.554**	0.595**	0.463**	0.591**	0.508**
CSC-W^a^	0.612**	0.378**	0.472**	0.358**	0.521**	0.398**	0.557**	0.419**	0.589**	0.476**	0.645**	0.538**	0.547**	0.420**	0.557**	0.450**
CaSUN-ES	0.452**	0.501**	0.386**	0.423**	0.523**	0.535**	0.476**	0.507**	0.520**	0.617**	0.233**	0.490**	0.442**	0.559**	0.548**	0.577**
CaSUN-CCC	0.304**	0.360**	0.184**	0.216**	0.320**	0.340**	0.335**	0.382**	0.279**	0.354**	0.156**	0.341**	0.245**	0.346**	0.396**	0.404**
CaSUN-INFO	0.318**	0.438**	0.200**	0.257**	0.315**	0.385**	0.280**	0.339**	0.254**	0.388**	0.108**	0.370**	0.223**	0.376**	0.402**	0.447**
CaSUN-QoL	0.403**	0.474**	0.306**	0.362**	0.432**	0.477**	0.412**	0.464**	0.406**	0.513**	0.208**	0.408**	0.364**	0.485**	0.477**	0.532**
CaSUN-REL	0.401**	0.501**	0.313**	0.409**	0.482**	0.565**	0.411**	0.449**	0.406**	0.523**	0.184**	0.437**	0.360**	0.515**	0.475**	0.518**
DASS-S	0.489**	0.415**	0.483**	0.444**	0.538**	0.509**	0.421**	0.398**	0.569**	0.547**	0.424**	0.508**	0.533**	0.501**	0.492**	0.505**
DASS-A	0.471**	0.491**	0.454**	0.472**	0.507**	0.524**	0.432**	0.403**	0.500**	0.514**	0.329**	0.485**	0.496**	0.522**	0.496**	0.493**
DASS-D	0.448**	0.372**	0.552**	0.506**	0.513**	0.498**	0.426**	0.415**	0.575**	0.564**	0.335**	0.468**	0.564**	0.517**	0.440**	0.458**
AQoL-IL	0.420**	0.369**	0.385**	0.367**	0.457**	0.398**	0.439**	0.419**	0.381**	0.384**	0.309**	0.422**	0.409**	0.397**	0.380**	0.337**
AQoL-REL	0.386**	0.335**	0.467**	0.452**	0.529**	0.457**	0.445**	0.400**	0.434**	0.406**	0.327**	0.400**	0.456**	0.398**	0.389**	0.382**
AQoL-MH	0.421**	0.333**	0.475**	0.434**	0.515**	0.455**	0.396**	0.418**	0.551**	0.530**	0.341**	0.455**	0.529**	0.527**	0.434**	0.434**
AQoL-COPING	0.396**	0.187**	0.450**	0.327**	0.429**	0.315**	0.384**	0.320**	0.484**	0.398**	0.377**	0.348**	0.468**	0.356**	0.327**	0.329**
AQoL-PAIN	0.395**	0.319**	0.370**	0.350**	0.392**	0.342**	0.358**	0.305**	0.352**	0.309**	0.312**	0.359**	0.362**	0.324**	0.286**	0.266**
AQoL-SEN	0.245**	0.254**	0.237**	0.227**	0.290**	0.286**	0.257**	0.236**	0.218**	0.206**	0.266**	0.303**	0.295**	0.234**	0.260**	0.225**
AQoL-OVERALL	0.494**	0.400**	0.516**	0.467**	0.569**	0.496**	0.495**	0.460**	0.527**	0.489**	0.429**	0.509**	0.556**	0.494**	0.460**	0.435**

**Correlation is significant at the 0.01 level. DIFF = Difficulties. UN = Unmet Needs. ERA = Executing Regular Actives, FMD = Finding Meaning and Enjoyment in Activities, RD = Relational Difficulties, OVF = Occupational/Vocational Functioning, PC = Psychological Challenges, VCD = Verbal Communication Difficulties, SFW = Social and Functioning and Withdrawal, IN = Informational Needs. PROMIS-COG = Patient-Reported Outcomes Measurement Information System-Cognitive Function Scale, CSC-W = Cognitive Symptom Checklist-Work, CaSUN-ES = Cancer Survivors Unmet Needs Scale-Existential Survivorship, CaSUN-CCC = Cancer Survivors Unmet Needs Scale-Comprehensive Cancer Care, CaSUN-INFO = Cancer Survivors Unmet Needs Scale-Information, CaSUN-QoL = Cancer Survivors Unmet Needs Scale-Information, CaSUN-REL = Cancer Survivors Unmet Needs Scale-Relationships, DASS-S = Depression, Anxiety, and Stress Scale-Stress, DASS-A = Depression, Anxiety, and Stress Scale-Anxiety, DASS-D = Depression, Anxiety, and Stress Scale-Depression, AQoL-IL = Assessment of Quality of Life-Independent Living, AQoL-REL = Assessment of Quality of Life-Relationships, AQoL-MH = Assessment of Quality of Life-Mental Health, AQoL-SEN = Assessment of Quality of Life-Senses. ^a^ CSC-W was only completed by participants currently in employment

### Discriminant validity

As expected, the strength of associations between domain theoretically-related MASCC COG-IMPACT subscales and the established measures scores consistently exceeded that of lesser theoretically-related domains. The two strongest and two weakest associations for each MASCC COG-IMPACT subscale across the “difficulties” and “unmet needs” indices are provided in Table 6. Furthermore, as expected, no associations between the MASCC COG-IMPACT subscales (across both “difficulties” and “unmet needs” indices) and the AQoL-Senses subscale exceeded a “small” correlation (*r* > 0.4; ranging from *r* = 0.206 to 0.303; see Table 5). Overall, the MASCC COG-IMPACT demonstrated strong discriminant validity across its subscales and indices.

**Table 6:** Discriminant validity associations

MASCC COG-IMPACT Subscale	Indices	Two Strongest Associations	Two Weakest Associations
Psychological Challenges			
	*Difficulties*	• PROMIS-COG (*r* = 0.661) • CSC-W (*r* = 0.589)	• AQoL-Senses (*r* = 0.218) • CaSUN-Information (*r* = 0.254)
	*Unmet Needs*	• CaSUN-ES (*r* = 0.617) • DASS-Depression (*r* = 0.598)	• AQoL-Senses (*r* = 0.206) • AQoL-Pain (*r* = 0.309)
Executing Regular Activities			
	*Difficulties*	• PROMIS-COG (*r* = 0.658) • CSC-W (*r* = 0.612)	• AQoL-Senses (*r* = 0.245) • CaSUN-CCC (*r* = 0.304)
	*Unmet Needs*	• CaSUN-ES (*r* = 0.501) • DASS-D (*r* = 0.491)	• AQoL-Coping (*r* = 0.187) • AQoL-Senses (*r* = 0.254)
Occupational/Vocational Functioning			
	*Difficulties*	• PROMIS-COG (*r* = 0.576) • CSC-W (*r* = 0.557)	• AQoL-Senses (*r* = 0.257) • CaSUN-Information (*r* = 0.280)
	*Unmet Needs*	• CaSUN-ES (*r* = 0.507) • CaSUN-QoL (*r* = 0.464)	• AQoL-Senses (*r* = 0.236) • AQoL-Pain (*r* = 0.305)
Informational Needs			
	*Difficulties*	• PROMIS-COG (*r* = 0.591) • CSC-W (*r* = 0.557)	• AQoL-Senses (*r* = 0.260) • AQoL-Pain (*r* = 0.286)
	*Unmet Needs*	• CaSUN-ES (*r* = 0.577) • CaSUN-Relationships (*r* = 0.518)	• AQoL-Senses (*r* = 0.225) • AQoL-Pain (*r* = 0.266)
Relational Difficulties			
	*Difficulties*	• PROMIS-COG (*r* = 0.573) • AQoL-Overall (*r* = 0.569)	• AQoL-Senses (*r* = 0.290) • CaSUN-Information (*r* = 0.315)
	*Unmet Needs*	• CaSUN-Relationships (*r* = 0.565) • CaSUN-ES (*r* = 0.535)	• AQoL-Senses (*r* = 0.286) • AQoL-Coping (*r* = 0.315)
Verbal Communication Challenges			
	*Difficulties*	• CSC-W (*r* = 0.645) • PROMIS-COG (*r* = 0.639)	• CaSUN-Information (*r* = 0.108) • CaSUN-CCC (*r* = 0.156)
	*Unmet Needs*	• PROMIS-COG (*r* = 0.554) • CSC-W (*r* = 0.538)	• AQoL-Senses (*r* = 0.303) • CaSUN-CCC (*r* = 0.341)
Finding Meaning and Enjoyment in Activities			
	*Difficulties*	• DASS-Depression (*r* = 0.552) • AQoL-Overall (*r* = 0.561)	• CaSUN-CCC (*r* = 0.184) • CaSUN-Information (*r* = 0.200)
	*Unmet Needs*	• DASS-Depression (*r* = 0.506) • DASS-Anxiety (*r* = 0.472)	• AQoL-Coping (*r* = 0.187) • AQoL-Senses (*r* = 0.254)
Social Functioning and Withdrawal			
	*Difficulties*	• PROMIS-COG (*r* = 0.595) • DASS-Depression (*r* = 0.639)	• CaSUN-Information (*r* = 0.223) • CaSUN-CCC (*r* = 0.245)
	*Unmet Needs*	• CaSUN-ES (*r* = 0.559) • DASS-Anxiety (*r* = 0.522)	• AQoL-Senses (*r* = 0.234) • AQoL-Pain (*r* = 0.324)

*PROMIS-COG* Patient-Reported Outcomes Measurement Information System-Cognitive Function Scale, *CSC-W* Cognitive Symptom Checklist-Work, *CaSUN-ES* Cancer Survivors Unmet Needs Scale-Existential Survivorship, *CaSUN-CCC* Cancer Survivors Unmet Needs Scale-Comprehensive Cancer Care. *CaSUN-QoL* Cancer Survivors Unmet Needs Scale-Information, *DASS* Depression, Anxiety, and Stress Scale

### Acceptability, appropriateness, and feasibility

The MASCC COG-IMPACT was rated as highly acceptable (M = 4.03, SD = 0.629), appropriate (M = 4.12, SD = 0.619), and feasible (M = 4.12, SD = 0.604), with all mean scores > 4 with five reflecting the highest possible score. Furthermore, 100% of the 55 MASCC COG-IMPACT items were endorsed as a “difficulty” for > 10% of participants, and the overall difficulties item endorsement was 51.52%. Overall, the MASCC COG-IMPACT was shown to be highly acceptable, appropriate and feasible.

### Final COG-IMPACT

The final MASCC COG-IMPACT is a 55-item, eight subscale, highly reliable, valid, acceptable, appropriate, and feasible self-report measure of difficulties and unmet needs related to CRCI. The MASCC COG-IMPACT protocol [9], initial qualitative phase [5], fillable PDF tool, Qualtrics tool, manual and scoring procedure, and automatic scoring sheet, are freely available for download and use (for non-commercial purposes) via the MASCC COG-IMPACT Open Science Framework Project Page (https://osf.io/5zc3a/), or via the Multinational Association for Supportive Care in Cancer (MASCC) webpage (https://mascc.org/resources/assessment-tools/).

## Discussion

Development and initial validation of the first purpose-built unmet needs assessment for CRCI: *Multinational Association of Supportive Care in Cancer - Unmet Needs Assessment of Cancer-Related Cognitive Impairment Impact (MASCC COG-IMPACT)* has been detailed. Existing unmet needs assessments in oncology and haematology usually focus on either very broad or very narrow domains, and usually not in the specific context of CRCI [22, 23, 45]. In contrast, the MASCC COG-IMPACT provides a fit-for-purpose unmet needs assessment that assesses the difficulties and unmet needs directly related to CRCI, thus supporting health professionals to provide highly individualised and optimal care to cancer survivors, that facilitates appropriate referral, support service provision, guide further assessment, and facilitate clinical and healthcare discussions.

Bonevski et al. [46] provided six criteria for guiding the determination of an oncology unmet needs assessment tool as effective: (1) the tool measures the multidimensional impact of cancer on cancer survivor needs, (2) directly and comprehensively assesses subjective health-related needs for help and support, (3) measures outcomes in a pre-defined temporal context, (4) demonstrates acceptable reliability and validity, (5) is user-friendly for all stakeholders, and (6) system-friendly. The MASCC COG-IMPACT strongly satisfies these criteria by measuring multiple domains impacted by CRCI, comprehensively assessing unmet needs across these domains, assessing difficulties and unmet needs within the specific context of CRCI, and within a specific timeframe, by having strong reliability and validity, being user-friendly due to co-design with cancer survivors and oncology health professionals, and by being system-friendly, allowing for its use across paper-based and electronic formats.

The multistep iterative approach to designing the MASCC COG-IMPACT has resulted in strong synergy between the findings within the qualitative [5] and quantitative phases of the project, with the extracted factors strongly reflecting the qualitative themes and subthemes. No items were required to be removed following the factor analysis, facilitating the strong content and face validity developed during the modified Delphi and cognitive interviewing phases, and further emphasising the methodological strengths of the measurement development approach [9]. The measurement development approach also resulted in the internal consistency of MASCC COG-IMPACT subscales exceeding that commonly found for other established unmet needs assessments in oncology. Many established unmet needs assessments used in cancer care include subscales which show “poor” internal consistencies of 0.5 to 0.6 [45, 47]. All internal consistencies of the MASCC COG-IMPACT subscales for the “difficulties” indices showed “good” or “very good” internal consistency (all ≥ 0.74) and all subscales for the “unmet needs” indices showed either “very good” or “excellent” internal consistency (all ≥ 0.87). Tian et al.’s [45] systematic review of the psychometric properties of unmet needs assessments in cancer found that less than half of unmet needs assessments assessed test–retest reliability, and only 30% of these found positive test–retest reliability (defined as ICC ≥ 0.70). The COG-IMPACT’s “difficulties” indices had an average test–retest reliability of 0.814 and the “unmet needs” indices had an average test–retest reliability of 0.785 over a 2-week retest interval, further demonstrating its strong reliability.

In clinical practice, the MASCC COG-IMPACT should be used in concert with subjective and objective assessments of cognition. This will facilitate the identification of CRCI and its severity, followed by an evaluation of unique and unresolved unmet needs associated with CRCI for a given person. The use of a subjective and objective assessment of cognition may precede the use of the MASCC COG-IMPACT. For example, an oncology health professional may use subjective (e.g., PROMIS-COG [38], FACT-COG [48], etc.) and objective (The Fast Cognitive Evaluation [49], Cambridge Neuropsychological Test Automated Battery [50], etc.) assessments to facilitate the determination of the presence and severity of CRCI, followed by the administration of the MASCC COG-IMPACT, if CRCI is determined, to understand the cancer survivor’s difficulties and unmet needs directly relating to their CRCI. The results of the MASCC COG-IMPACT administration can then be used to facilitate optimal person-centred care and referral in line with a cancer survivor’s CRCI-related difficulties and unmet needs [5]. In research, the MASCC COG-IMPACT may be used to explore (a) what clinical and demographic characteristics contribute toward CRCI-related difficulties and unmet needs, (b) how interventions can impact CRCI-related difficulties and unmet needs, and (c) how specific domains of CRCI-related difficulties and unmet needs can predict clinical and psychosocial outcomes, for example, return-to-work [51], or the development of psychopathology [3, 52, 53]. The MASCC COG-IMPACT may also be used by cancer survivors to elucidate their difficulties and supportive needs and advocate for their care and further research.

### Strengths, limitations and directions for future research

This measurement development project had a number of strengths. The multistep iterative measurement development approach was highly rigorous, featuring a strong emphasis on co-design with both cancer survivors and health professionals. The mixed methods approach allowed for a “bottom-up” development of the measure of acknowledging lived experience, as well as psychometric validity. Furthermore, the cancer survivor and health professional samples across the steps were international and considered a wide variety of clinical and demographic characteristics and lived experiences. Lastly, the specific methodologies and analytical techniques utilised throughout the project were in line with gold standard recommendations for best practice.

This project did however have some limitations. This initial development and validation of the MASCC COG-IMPACT was conducted in adult cancer survivors who have undergone, and completed, successful curative-intent cancer treatment, letting its applicability currently to only this population. Future research should further develop and validate the MASCC COG-IMPACT in other cancer cohorts, such as those currently undergoing treatment for curative intent, advanced and metastatic cancer survivors, and adolescents and young adults. Further, time since active treatment completion of participants within step #7 was not obtained, thereby limiting our understanding of this sample’s characteristics. Also, the majority of the participants throughout the steps of the project were female. While this is common within cancer survivorship research, it may have impacted the findings. Future research should further examine the reliability and validity of the MASCC COG-IMAPCT, using mixed methods approaches within and between different sexes and genders. Additionally, while the MASCC COG-IMPACT is valid and reliable, it is only currently available in English, and it may be found to be too lengthy for some clinical and research contexts. Future research should develop and validate other language versions that are culturally adapted to their given context as well as develop and validate a short-form version of the MASCC COG-IMPACT. Finally, future research should seek to develop a care pathway to facilitate consistent and evidence-based care informed by data collected from the MASCC COG-IMPACT.

## Supplementary Information

Below is the link to the electronic supplementary material.

MASCC COG-IMPACT Automatic Scoring Sheet Electronic Supplementary file 1 (XLSX 55 KB)

MASCC COG-IMPACT Qualtrics File Electronic Supplementary file 2 (QSF 266 KB)

MASCC COG-IMPACT Manual and Scoring Procedure Electronic Supplementary file 3 (PDF 2.15 MB)

MASCC COG-IMPACT Tool Fillable and Printable Pdf Electronic Supplementary file 4 (PDF 1.87 MB)

MASCC COG-IMPACT Qualtrics Use Instructions Electronic Supplementary file 5 (TXT 0 KB)

Supplementary Data Electronic Supplementary file 6 (PDF 343 KB)

## Data Availability

The data may be made available on request of the corresponding author and to the satisfaction of the granting HREC.
