# Supplementary material for: The MASCC COG-IMPACT: An unmet needs assessment for cancer-related cognitive impairment impact developed by the Multinational Association of Supportive Care in Cancer
Source: Support Care Cancer. 2025 Jan 24;33(2):120. doi: 10.1007/s00520-025-09149-7 (PMC11761510; doi:10.1007/s00520-025-09149-7)
Supplement: Supplementary file 4 — Electronic Supplementary file 4 (PDF 1.87 MB) [file 520_2025_9149_MOESM4_ESM.pdf]

MASCC™ COG-IMPACT

Unmet Needs Assessment of Cancer-Related Cognitive Impairment Impact

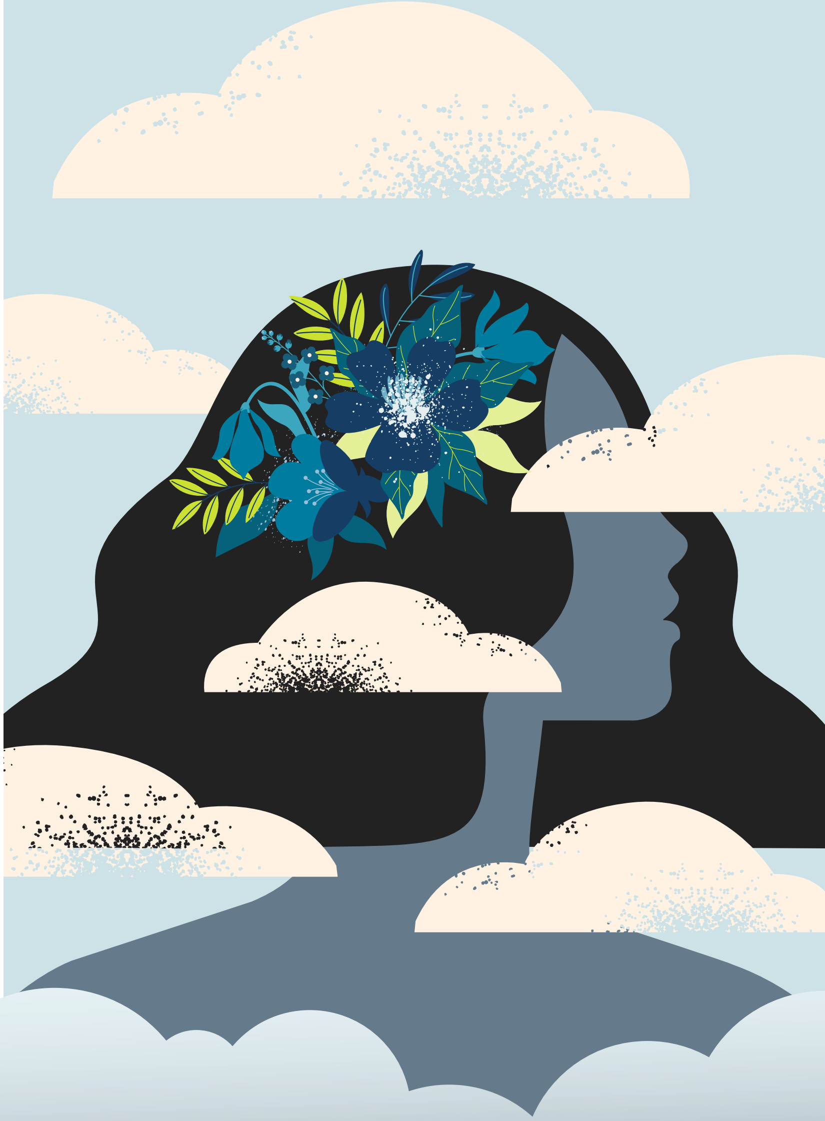

The MASCC COG-IMPACT

An official tool of the Multinational Association of Supportive Care in Cancer (MASCC.ORG)

Version 1.0

©Multinational Association of Supportive Care in Cancer (MASCC). All Rights Reserved Worldwide.

Instructions

Cancer-related cognitive impairment (CRCI) refers to a common side effect of cancer and its treatments. It can lead to changes in cognitive abilities, such as thinking clearly, remembering, concentrating, planning, engaging in conversations, and performing daily tasks.

The purpose of this assessment tool is to understand the difficulties that you've faced, and any unmet supportive care needs related to your experience of living with CRCI over the last month.

For each item, please select 'No' or 'Yes' indicating if you experienced the difficulty. If 'Yes' then please select the response that best describes how you feel that you have been in need of help or support regarding the CRCI-related issue **OVER THE LAST MONTH**.
The response options are described below:

1. I **do not** need any additional support = applicable to me but I don't need help or support for this issue
2. My need for support is **satisfied** = I experience this difficulty, but I receive adequate help or support
3. I have a **low need** for additional support = I experience this difficulty and need some help or support
4. I have a **moderate need** for additional support = I experience this difficulty and need a moderate amount of help or support
5. I have a **high need** for additional support = I experience this difficulty and need a high amount of help or support

Example

If you select '**YES**' and then choose '**I have a moderate need for additional support**' as below, it indicates that you are experiencing this difficulty and that you have a moderate need for help or support in managing or coping with the difficulty. This assistance could come from a health professional such as a psychologist, occupational therapist, or counsellor, or a family member or caregiver.

Experiencing the difficulty does not necessarily mean that you have unmet support needs. For example, you might occasionally forget things that you need (like your keys, wallet, or phone) but you have developed effective strategies to manage this, and therefore do not require further help or support. In this case, you would select '**YES**' and '**My need for support is satisfied**.'

**Your CRCI related Difficulties**<br>In the last month, ***because of your CRCI***, did you experience any of the following difficulties:			**Your Unmet Needs**<br>**If yes, how much additional support do you need?**					
			I **do not** need any additional support	My need for support is **satisfied**	I have a **low need** for additional support	I have a **moderate need** for additional support	I have a **high need** for additional support	RESET
Because of my CRCI...<br>I have stopped or reduced doing the things I enjoy	○ NO	● YES	○ 1	○ 2	○ 3	● 4	○ 5	

©Multinational Association of Supportive Care in Cancer (MASCC). All Rights Reserved Worldwide.

Executing Regular Activities

**Your CRCI related Difficulties**<br>In the last month, ***because of your CRCI***, did you experience any of the following difficulties:			**Your Unmet Needs**<br>**If yes**, how much additional support do you need?<br>I **do not** need any additional support	My need for support is **satisfied**	I have a **low need** for additional support	I have a **moderate need** for additional support	I have a **high need** for additional support	RESET
Because of my CRCI...<br>I forget things I need (e.g. keys, wallet, phone)	NO	YES	1	2	3	4	5	
Because of my CRCI...<br>I have difficulty remembering what I intend to do in my day	NO	YES	1	2	3	4	5	
Because of my CRCI...<br>I have to make more of an effort to perform my daily tasks	NO	YES	1	2	3	4	5	
Because of my CRCI...<br>I often forget things I need in life (e.g. pin number for cards, passwords, email addresses)	NO	YES	1	2	3	4	5	
Because of my CRCI...<br>I often forget instructions health professionals have given me (e.g. to exercise at certain times, take medication)	NO	YES	1	2	3	4	5	
Because of my CRCI...<br>I have stopped doing things I enjoy that require too much mental effort (e.g. puzzles, crosswords)	NO	YES	1	2	3	4	5	
Because of my CRCI...<br>I have trouble remembering important events for my partner/family members/friends	NO	YES	1	2	3	4	5	

©Multinational Association of Supportive Care in Cancer (MASCC). All Rights Reserved Worldwide.

Finding Meaning and Enjoyment in Activities

**Your CRCI related Difficulties** In the last month, ***because of your CRCI***, did you experience any of the following difficulties:			**Your Unmet Needs** **If yes**, how much additional support do you need? I **do not** need any additional support	My need for support is **satisfied**	I have a **low need** for additional support	I have a **moderate need** for additional support	I have a **high need** for additional support	RESET
Because of my CRCI... I have stopped or reduced doing the things I enjoy	NO	YES	1	2	3	4	5	
Because of my CRCI... I am having trouble engaging with things I find meaningful	NO	YES	1	2	3	4	5	
Because of my CRCI... I have trouble enjoying things I used to enjoy	NO	YES	1	2	3	4	5	
Because of my CRCI... I have trouble engaging in hobbies I enjoy	NO	YES	1	2	3	4	5	

©Multinational Association of Supportive Care in Cancer (MASCC). All Rights Reserved Worldwide.

Relational Difficulties

**Your CRCI related Difficulties** In the last month, ***because of your CRCI***, did you experience any of the following difficulties:		**Your Unmet Needs** **If yes**, how much additional support do you need? →	I **do not** need any additional support	My need for support is **satisfied**	I have a **low need** for additional support	I have a **moderate need** for additional support	I have a **high need** for additional support	RESET
Because of my CRCI... I have trouble keeping up with the requirements of being a partner/family member/friend	NO	YES	1	2	3	4	5	
Because of my CRCI... I am concerned that a partner/family member/friend will joke about my forgetfulness	NO	YES	1	2	3	4	5	
Because of my CRCI... I have trouble managing the pressures of being a partner/family member/friend	NO	YES	1	2	3	4	5	
Because of my CRCI... My partner/family members/friends have difficulty understanding some things I do or struggle with	NO	YES	1	2	3	4	5	
Because of my CRCI... There is a change in the dynamic of my relationship(s) with my partner/family members/friends	NO	YES	1	2	3	4	5	
Because of my CRCI... Changes to my emotional state has impacted my relationships with my partner/family members/friends	NO	YES	1	2	3	4	5	
Because of my CRCI... I feel I am a burden to my partner/family members/friends	NO	YES	1	2	3	4	5	
Because of my CRCI... I struggle with feelings of guilt because of the impact on my partner/family members/friends	NO	YES	1	2	3	4	5	
Because of my CRCI... My partner/family members/friends has taken on more at home (e.g. daily tasks, financial decisions)	NO	YES	1	2	3	4	5	

©Multinational Association of Supportive Care in Cancer (MASCC). All Rights Reserved Worldwide.

Occupational/Vocational Functioning

**Your CRCI related Difficulties**<br>In the last month, ***because of your CRCI***, did you experience any of the following difficulties:<br>*Note.* "school" refers to any educational setting. For example, University, College, Highschool, etc.			I **do not** need any additional support	My need for support is **satisfied**	I have a **low need** for additional support	I have a **moderate need** for additional support	I have a **high need** for additional support	RESET
		**Your Unmet Needs**<br>**If yes**, how much additional support do you need?						
Because of my CRCI...<br>I struggle with feeling ready to work/volunteer/school	NO	YES	1	2	3	4	5	
Because of my CRCI...<br>I need specific strategies to feel more comfortable returning to work/volunteering/school	NO	YES	1	2	3	4	5	
Because of my CRCI...<br>I do not know what my working/volunteering/schooling capacity is	NO	YES	1	2	3	4	5	
Because of my CRCI...<br>I am concerned about letting others down at work/volunteering/school	NO	YES	1	2	3	4	5	
Because of my CRCI...<br>I need to understand what changes are required to return to work/volunteering/school (e.g. increased breaks, shorter days)	NO	YES	1	2	3	4	5	
Because of my CRCI...<br>I feel I cannot work/volunteer/school at my previous capacity	NO	YES	1	2	3	4	5	
Because of my CRCI...<br>Others perceive me differently at work/volunteering/school	NO	YES	1	2	3	4	5	
Because of my CRCI...<br>I have difficulty understanding complex ideas, concepts or processes at work/volunteering/school	NO	YES	1	2	3	4	5	
Because of my CRCI...<br>I need some accommodations at work/volunteering/school to better cope	NO	YES	1	2	3	4	5	
Because of my CRCI...<br>I have difficulty with certain tasks at work/volunteering/school	NO	YES	1	2	3	4	5	

©Multinational Association of Supportive Care in Cancer (MASCC). All Rights Reserved Worldwide.

Psychological Challenges

**Your CRCI related Difficulties**<br>In the last month, ***because of your CRCI***, did you experience any of the following difficulties:		**Your Unmet Needs**<br>**If yes**, how much additional support do you need?					
			I **do not** need any additional support	My need for support is **satisfied**	I have a **low need** for additional support	I have a **moderate need** for additional support	I have a **high need** for additional support	RESET
Because of my CRCI...<br>I feel like a different person compared to who I was before cancer	NO	YES	1	2	3	4	5	
Because of my CRCI...<br>I feel unsure of myself and my abilities	NO	YES	1	2	3	4	5	
Because of my CRCI...<br>I feel that I have lost my self-confidence	NO	YES	1	2	3	4	5	
Because of my CRCI...<br>I struggle to feel confident in my ability to cope with life's challenges	NO	YES	1	2	3	4	5	
Because of my CRCI...<br>I feel I can't trust myself	NO	YES	1	2	3	4	5	
Because of my CRCI...<br>I am feeling overwhelmed	NO	YES	1	2	3	4	5	
Because of my CRCI...<br>I often get frustrated when I cannot remember something	NO	YES	1	2	3	4	5	
Because of my CRCI...<br>I am struggling with anxiety	NO	YES	1	2	3	4	5	
Because of my CRCI...<br>I get frustrated as I am not good at things I used to be (e.g. certain tasks, puzzles, games)	NO	YES	1	2	3	4	5	
Because of my CRCI...<br>I struggle with a sense of loss for who I once was	NO	YES	1	2	3	4	5	

©Multinational Association of Supportive Care in Cancer (MASCC). All Rights Reserved Worldwide.

Verbal Communication Challenges

**Your CRCI related Difficulties**<br>In the last month, ***because of your CRCI***, did you experience any of the following difficulties:			**Your Unmet Needs**<br>**If yes**, how much additional support do you need?<br>I **do not** need any additional support	My need for support is **satisfied**	I have a **low need** for additional support	I have a **moderate need** for additional support	I have a **high need** for additional support	RESET
Because of my CRCI...<br>I cannot find words easily	NO	YES	1	2	3	4	5	
Because of my CRCI...<br>I lose my train of thought in a conversation	NO	YES	1	2	3	4	5	
Because of my CRCI...<br>I cannot remember details I should remember in a conversation (e.g. peoples' names, details about a friend's life)	NO	YES	1	2	3	4	5	
Because of my CRCI...<br>I often have to ask people to repeat themselves in conversation	NO	YES	1	2	3	4	5	

©Multinational Association of Supportive Care in Cancer (MASCC). All Rights Reserved Worldwide.

Social Functioning and Withdrawal

**Your CRCI related Difficulties** In the last month, ***because of your CRCI***, did you experience any of the following difficulties:			**Your Unmet Needs** **If yes**, how much additional support do you need? I **do not** need any additional support	My need for support is **satisfied**	I have a **low need** for additional support	I have a **moderate need** for additional support	I have a **high need** for additional support	RESET
Because of my CRCI... I tend to be more quiet than usual in group conversations	NO	YES	1	2	3	4	5	
Because of my CRCI... I tend to make excuses to get out of social interactions	NO	YES	1	2	3	4	5	
Because of my CRCI... I have withdrawn from social activities I used to enjoy	NO	YES	1	2	3	4	5	
Because of my CRCI... I am isolating myself from others	NO	YES	1	2	3	4	5	
Because of my CRCI... I feel anxious in social situations	NO	YES	1	2	3	4	5	
Because of my CRCI... I am drained of energy after social interactions	NO	YES	1	2	3	4	5	

©Multinational Association of Supportive Care in Cancer (MASCC). All Rights Reserved Worldwide.

Informational Needs

**Your CRCI related Difficulties** In the last month, ***because of your CRCI***, did you experience any of the following difficulties:		**Your Unmet Needs** **If yes**, how much additional support do you need?						
			I **do not** need any additional support	My need for support is **satisfied**	I have a **low need** for additional support	I have a **moderate need** for additional support	I have a **high need** for additional support	RESET
Because of my CRCI... I need to be informed about what things I can do to help myself manage or improve	NO	YES	1	2	3	4	5	
Because of my CRCI... I sometimes fear that I am losing my mind, going crazy, or that I am experiencing early signs of dementia	NO	YES	1	2	3	4	5	
Because of my CRCI... I need help finding or accessing resources (e.g. information sheets, brochures) that I can give to others (friends, family, employers) to help them understand CRCI	NO	YES	1	2	3	4	5	
Because of my CRCI... I need information about what to expect about CRCI (e.g. how long it will last, whether it changes with time)	NO	YES	1	2	3	4	5	
Because of my CRCI... I am not sure if my experience is normal	NO	YES	1	2	3	4	5	

©Multinational Association of Supportive Care in Cancer (MASCC). All Rights Reserved Worldwide.

For Health Professional Use - Scoring

Note that the item scoring does not match the numbered response options in the MASCC COG-IMPACT. The numbered response options in the tool are to facilitate usability only and are not to be used for scoring. Instead, use the scoring instructions on this page.

For further resources and guidance, including an automatic scoring file and how to handle missing data, access the MASCC COG-IMPACT Resources here: https://osf.io/5zc3a/

A '**Difficulties Subscale Score**' (**DSS**) of each of the 8 subscales of the MASCC COG-IMPACT may be calculated by summing the scores for all Difficulties items within each subscale, and dividing this Difficulties Subscale Total (**DST**) by the number of items within the particular subscale (**nSI**);

$$DSS = \frac{DST}{nSI}$$

Difficulties Subscale Scores can range from 0 to 1. Higher Difficulties Subscale Scores indicate a greater number of Difficulties.

An '**Unmet Needs Subscale Score**' (**UNSS**) of each of the 8 subscales of the MASCC COG-IMPACT may be calculated by summing the scores for all Unmet Needs items within each subscale, and dividing this Unmet Needs Subscale Total (**UNST**) by the number of items within the particular subscale (**nSI**);

$$UNSS = \frac{UNST}{nSI}$$

Unmet Needs Subscale Scores can range from 0 to 3. Higher subscale scores indicate a greater severity of Unmet Needs.

A '**Difficulties Total Score**' (**DTS**) may be calculated to provide an indication of overall difficulties across the subscales. The Difficulties Total Score may be calculated by summing all 8 MASCC COG-IMPACT difficulties subscale scores, and dividing this Difficulties Total (**DT**) by the number of subscales in the MASCC COG-IMPACT (**nSS**);

$$DTS = \frac{DT}{nSS}$$

nSS = 8

The Difficulties Total Score can range from 0 to 1.
A higher Difficulties Total Score indicates a greater number of Difficulties.

Difficulties Item Scoring:	
No	= 0
Yes	= 1
**Scoring Item Responses:**	
'**No**' to 'Difficulty' response option	= 0
I **do not** need any additional support	= 0
My need for support is **satisfied**	= 0
I have a **low need** for additional support	= 1
I have a **moderate need** for additional support	= 2
I have a **high need** for additional support	= 3

Number of Items in Each Subscale:	
nSI = 7	*Executing Regular Activities*
nSI = 4	*Finding Meaning and Enjoyment in Activities*
nSI = 9	*Relational Difficulties*
nSI = 10	*Occupational/Vocational Functioning*
nSI = 10	*Psychological Challenges*
nSI = 4	*Verbal Communication Challenges*
nSI = 6	*Social Functioning and Withdrawal*
nSI = 5	*Informational Needs*

A '**Unmet Needs Total Score**' (**UNTS**) may be calculated to provide an indication of overall Unmet Needs across all of the subscales. The Unmet Need Total Score may be calculated by summing all 8 MASCC COG-IMPACT unmet needs subscale scores, and dividing this Unmet Need Total (**UNT**) by the number of subscales in the MASCC COG-IMPACT (**nSS**);

$$UNTS = \frac{UNT}{nSS}$$

nSS = 8

The Unmet Need Total Score can range from 0 to 3.
A higher total Unmet Needs score indicates a greater severity of Unmet Needs.

©Multinational Association of Supportive Care in Cancer (MASCC). All Rights Reserved Worldwide.

For Health Professional Use

SCORING TABLE				$DSS = \frac{DST}{nSI}$	$UNSS = \frac{UNST}{nSI}$	$DTS = \frac{DT}{nSS}$	$UNTS = \frac{UNT}{nSS}$
Subscales		Difficulties Subscale Total (DST)	Unmet Needs Subscale Total (UNST)	Difficulties Subscale Score (DSS)	Unmet Needs Subscale Score (UNSS)	Difficulties Total Score (DTS)	Unmet Needs Total Score (UNTS)
nSI = 7	*Executing Regular Activities*						
nSI = 4	*Finding Meaning and Enjoyment in Activities*						
nSI = 9	*Relational Difficulties*						
nSI = 10	*Occupational/ Vocational Functioning*						
nSI = 10	*Psychological Challenges*						
nSI = 4	*Verbal Communication Challenges*						
nSI = 6	*Social Functioning and Withdrawal*						
nSI = 5	*Informational Needs*						
**Total Measure Scores:**							

Notes:

RESET FORM

©Multinational Association of Supportive Care in Cancer (MASCC). All Rights Reserved Worldwide.
