# Supplementary material for: The MASCC COG-IMPACT: An unmet needs assessment for cancer-related cognitive impairment impact developed by the Multinational Association of Supportive Care in Cancer
Source: Support Care Cancer. 2025 Jan 24;33(2):120. doi: 10.1007/s00520-025-09149-7 (PMC11761510; doi:10.1007/s00520-025-09149-7)
Supplement: Supplementary file 6 — Electronic Supplementary file 6 (PDF 343 KB) [file 520_2025_9149_MOESM6_ESM.pdf]

SUPPLEMENTARY MATERIALS

The MASCC COG-IMPACT: An Unmet Needs Assessment for Cancer-Related Cognitive Impairment Impact Developed by the Multinational Association of Supportive Care in Cancer

Supportive Care in Cancer

Online Resource Supplementary Materials

Darren Haywood PhD* [1,2,3,4], Alexandre Chan PharmD[5], Raymond J. Chan PhD[6], Frank D. Baughman PhD[4], Evan Dauer MClinPsych[1,2], Haryana M. Dhillon PhD[7], Ashley M. Henneghan PhD[8,9], Blake J. Lawrence PhD[4], Maryam B. Lustberg MD[10], Moira O'Connor PhD[4], Janette L. Vardy MD PhD[11], Susan L. Rossell[+] PhD[2,12] & Nicolas H. Hart[+] PhD [1,6,13,14,15]

[1] Human Performance Research Centre, INSIGHT Research Institute, Faculty of Health, University of Technology Sydney (UTS), NSW, Australia.
[2] Department of Mental Health, St Vincent's Hospital Melbourne, Fitzroy, VIC, Australia.
[3] Department of Psychiatry, Faculty of Medicine, Dentistry and Health Sciences, University of Melbourne, VIC, Australia.
[4] School of Population Health, Faculty of Health Sciences, Curtin University, Bentley, WA, Australia.
[5] School of Pharmacy and Pharmaceutical Sciences, University of California, Irvine, USA
[6] Caring Futures Institute, College of Nursing and Health Sciences, Flinders University, Adelaide, SA, Australia
[7] University of Sydney, Faculty of Science, School of Psychology, Psycho-Oncology Cooperative Research Group, Sydney, Australia
[8] School of Nursing, University of Texas at Austin, Austin, Texas, USA
[9] Department of Oncology, Dell Medical School, The University of Texas at Austin, Austin, Texas, USA
[10] Yale University School of Medicine, New Haven, CT, USA
[11] Faculty of Medicine and Health, The University of Sydney, Sydney, Australia
[12] Centre for Mental Health and Brain Sciences, Swinburne University of Technology, Hawthorn, VIC, Australia
[13] Cancer and Palliative Care Outcomes Centre, Faculty of Health, Queensland University of Technology (QUT), QLD, Australia.
[14] Exercise Medicine Research Institute, School of Medical and Health Science, Edith Cowan University, WA, Australia
[15] Institute for Health Research, University of Notre Dame Australia, WA, Australia

[+] SLR and NHH are joint senior authors

Corresponding Author:

Dr Darren Haywood, PhD, BPsych(Hons)
Postdoctoral Research Fellow (Cancer Survivorship), INSIGHT Research Institute | Faculty of Health
University of Technology Sydney, Moore Park, Sydney, NSW, 2030.
Darren.haywood@uts.edu.au

SUPPLEMENTARY MATERIALS

Table 1. Sample Characteristics for Delphi Participants

Characteristic	Mean (SD)/Range/%
**Age (years)**	M = 46.57 years (SD = 13.18, Min = 25, Max = 71)
**Gender**	
Male	5 (17.2%)
Female	24 (82.8%)
**Country of Residence**	
Australia	10 (34.5%)
USA	7 (24.1%)
Canada	2 (6.9%)
Croatia	1 (3.4%)
Egypt	1 (3.4%)
Hong Kong	1 (3.4%)
India	1 (3.4%)
Japan	1 (3.4%)
Mexico	1 (3.4%)
Portugal	1 (3.4%)
The Netherlands	1 (3.4%)
Turkey	1 (3.4%)
UK	1 (3.4%)
**Primary Discipline**	
Radiation Oncologist	1 (3.4%)
Medical Oncologist	4 (13.8%)
Haematologist	2 (6.9%)
Nurse	8 (27.6%)
Physiotherapist or Occupational Therapist	6 (20.7%)
Psychologist	3 (10.3%)
Academic Researcher	13 (44.8%)
Primary Care Physician	3 (10.3%)
Palliative Care Physician	1 (3.4%)
Social Worker	1 (3.4%)
**Highest level of Education**	
Bachelor’s degree	7 (24.1%)
Master’s Degree	5 (17.2%)
MD or MBSS	5 (17.2%)
PhD	12 (41.4%)
**Years in Oncology**	M = 17.14 years (SD = 10.52, Min = 1, Max = 40)
**Primary Cancer Sites in Role**	
Breast	20 (69.0%)
Colorectal	18 (62.1%)
Gynaecological	13 (44.8%)
Bone	10 (34.5%)
CNS	14 (48.3%)
Lung	13 (44.8%)
Neck	13 (44.8%)
Prostate	13 (44.8%)
Haematological	14 (48.3%)
Lymphoma	15 (51.7%)
Site Unknown	7 (24.1%)
Other	7 (27.1%)

SUPPLEMENTARY MATERIALS

Supplementary Table 2. Delphi Survey Responses	
**Theme 1 (from steps #1 and #2): Executing regular activities**<br><br>**Subtheme 1 (from steps #1 and #2: Difficulties in daily tasks**	
**Item**	**N Endorsement**
I forget things I need (e.g. keys, wallet, phone)	19
I have difficulty remembering what I intend to do in my day	15
I feel I have to make more of an effort to perform my daily tasks	14
I often forget important things I need in life (e.g. pin number for cards, passwords, email addresses)	13
I often forget instructions health professionals have given me (e.g. to exercise at certain times, take medication)	13
I need to implement specific strategies to help me cope with forgetfulness	12
I struggle with completing household tasks	8
have trouble remembering to complete tasks around the house (e.g. cooking, cleaning)	7
I have trouble remembering to take my daily medication	7
I have trouble remembering items I need when I go shopping	6
I have trouble finding my way to or from a familiar place	6
I have difficulty cooking meals that were once very easy for me	5
I lack confidence driving my car anywhere by myself	4
I have trouble following a recipe	2
I need support to feel comfortable driving	2
I have stopped driving	2
I need support to feel comfortable travelling on public transport (e.g. buses, trams, trains)	1
I lack confidence navigating my way home from a new area	1
I have trouble feeling alert when I am driving	1
**Theme 1: Executing regular activities**<br><br>**Subtheme 2: Difficulty engaging in valued activities**	
**Item**	**N Endorsement**
I have stopped doing things I enjoy that require too much mental effort (e.g. puzzles, crosswords)	22
I have stopped or reduced doing the things I enjoy	19
I am having trouble engaging with things I find meaningful	17
I have trouble enjoying things I used to enjoy	15
I have trouble engaging in hobbies I enjoy	14
I am having trouble finding enjoyment in things I used to enjoy	13
I am no longer doing things I enjoy	11
I am having trouble engaging in creative pursuits (e.g. drawing, painting, music) that I used to enjoy	11
I have reduced or stopped reading for pleasure	9
I do not get the same enjoyment I once did from my hobbies	5
I do not engage in reading for the enjoyment of it	3
I have reduced or stopped playing musical instruments	1
**Theme 2 (from steps #1 and #2): Relational Difficulties**<br><br>**Subtheme 1 (from steps #1 and #2): Difficulty parenting**	
**Item**	**N Endorsement**
I have trouble remembering important events for my children (e.g. appointments, extra-curricular activities, sporting practice)	21
I have trouble keeping up with the requirements of being a parent	20
I feel that I am not coping with the demands of being a parent	18
I have trouble managing the stress of parenting	16
My children have difficulty understanding some things I do or struggle with	13
I struggle to cope with the everyday frustrations that come with children	12
I feel there is a strain on my relationship with my child/children	11
I have trouble connecting with my child/children	9
My children are frustrated with me	8
My children need support for their own mental health	7
**Theme 2: Relational difficulties**	

SUPPLEMENTARY MATERIALS

**Subtheme 2: Impacts on intimate relationships**	
**Item**	**N Endorsement**
There is a change in the dynamic of my relationship/s	14
My own psychological distress has impacted my relationships (with partner, children, friends)	12
I feel I am a burden to my partner	11
I struggle with feelings of guilt because of the impact on my partner	11
My partner has taken on more at home (e.g. daily tasks, financial decisions)	10
I have difficulty developing new intimate relationships	10
My partner needs help understanding CRCI and how to cope with it	10
I struggle to make decisions without the input or help from my partner	9
I struggle with the idea that I am not the person I was when I met my partner	8
My intimate partner is becoming irritated or frustrated with me	7
I have difficulty managing the stress within my intimate relationship	6
I struggle with feeling worthy or good enough for my partner	6
My partner has to remember things for me (e.g. medications to take, appointments, birthdays)	5
I struggle managing the shift/change in roles at home	4
I have trouble connecting with my partner	4
I feel my partner is carrying a bigger load	3
I find dating difficult	3
I am worried about how I will go with the dating process	3
I find making friends difficult	2
I react to little things my partner does	2
**Theme 3 (from steps #1 and #2): Occupational functioning**<br><br>**Subtheme 1 (from steps #1 and #2): Difficulty in returning to work**	
**Item**	**N Endorsement**
The thought of returning to work is causing me distress (e.g. anxiety, worry, concern)	20
I need specific strategies to feel more comfortable returning to work	14
I do not know what my working capacity is	14
I worry that I would let others down at work	13
I feel I need to understand what I need in place to return to work (e.g. increased breaks, shorter days)	11
I struggle with feeling ready to return to work	10
I feel embarrassed to ask for a gradual return to work	10
I feel I need support from my employer or colleagues to return to work	10
I am not confident that I could cope if I returned to work	10
I do not feel I have the capacity to return to work	9
I feel reluctant to return to work	9
I feel I need help from a professional to help me to return to work	8
I feel I need support from my employer before I return to work	2
**Theme 3 (from steps #1 and #2): Occupational functioning**<br><br>**Subtheme 2 (from steps #1 and #2): Decreased work capacity**	
**Item**	**N Endorsement**
I feel I cannot work at my previous capacity	18
I worry about how others perceive me at work	13
I have difficulty understanding complex ideas, concepts or processes at work	11
I feel I need some accommodations at work to better cope	9
I have difficulty with certain tasks at work	8
I cannot seem to do what were once simple tasks at work	7
I cannot focus at work	7
I feel it is more effortful to perform my work duties	7
I need help with making my employer and/or colleagues understand CRCI	6
I have considered reducing my hours at work	6
I cannot multi-task at work	6

SUPPLEMENTARY MATERIALS

I am less productive at work	6
I do not feel confident completing tasks at work	5
I feel incompetent at work	5
I avoid taking on too much at work	5
I feel I can only take on tasks at work that are easy and very familiar to me	5
I have considered resigning from work	4
I have difficulty understanding new or unfamiliar ideas or concepts at work	4
I have difficulty remembering instructions at work	3
I avoid difficult tasks or projects at work	2
I am struggling at work	2
I feel I need to communicate to my employer and/or colleagues about CRCI	0
I prefer to only do tasks that are repetitive at work	0
I have forgotten how to do basic tasks at work	0
**Theme 4 (from steps #1 and #2): Psychological distress**<br><br>**Subtheme 1 (from steps #1 and #2): Loss of confidence**	
**Item**	**N Endorsement**
I feel like a different person compared to who I was before cancer treatment	22
I feel unsure of myself and abilities	20
I feel that I have lost my self-confidence	15
I struggle to feel confident in my ability to cope with life's challenges.	12
I feel I can't trust myself	12
I struggle with feeling confident in myself	12
I doubt myself often	11
I do not feel as effective in my life	10
I do not feel confident I could cope with difficult situations	9
I feel I need to build my confidence back up	7
I struggle with taking on too much for lack of confidence I can handle it	6
I do not feel comfortable with the person I am	3
**Theme 4 (from steps #1 and #2): Psychological distress**<br><br>**Subtheme 2 (from steps #1 and #2): Frustration and decreased distress tolerance**	
**Item**	**N Endorsement**
I am feeling overwhelmed	14
I often get frustrated when I cannot remember something	11
I am struggling with anxiety	10
I get frustrated as I am not good at things I used to be (e.g. certain tasks, puzzles, games)	10
I struggle with a sense of loss for who I once was	9
I sometimes get emotional for no particular reason	8
I am struggling with my mental health	8
I get frustrated at my decreased capacity to cope with life's challenges	8
I get frustrated over small things	7
I feel my tolerance to cope with life's challenges has lowered	7
I have a decreased sense of enthusiasm or excitement for life	6
I am struggling with a lack of purpose in life	6
I can become emotional by minor things	5
I feel disappointed in myself	5
I struggle with feelings of embarrassment	5
I get bothered by things that usually would not bother me	4
I am struggling with depression	4
I am struggling with feeling low	4
I am struggling with identity change	4
I cannot seem to manage my frustration	3
I can get frustrated in conversations with others	2
I am having trouble being grateful in my life	0
**Theme 5 (from steps #1 and #2): Social functioning**	

SUPPLEMENTARY MATERIALS

**Subtheme 1 (from steps #1 and #2): Difficulty in conversation**	
**Item**	**N Endorsement**
I cannot find words easily	16
I lose my train of thought in a conversation	15
I cannot remember details I should remember in a conversation (e.g. peoples names, details about a friends life)	12
I tend to be more quiet than usual in group conversations	12
I often have to ask people to repeat themselves in conversation	9
I struggle to convey what I would like to in a conversation	8
I find it hard to follow group conversations	8
I struggle to keep up in a group conversation	7
I am not as assertive in conversation	7
I lose track of the topic of conversation	6
Some things I say do not come out how I would like them to	6
I have difficulty following group conversations	6
I have difficulty remembering the content of a conversation	6
I lose my words in a conversation	5
I feel embarrassed because I have forgotten something in conversation	5
I feel uncomfortable when interacting with others	4
I have trouble connecting with others in conversation	4
I forget parts of a conversation	3
**Theme 5 (from steps #1 and #2): Social functioning**<br><br>**Subtheme 2 (from steps #1 and #2): Social avoidance**	
**Item**	**N Endorsement**
I feel alone in my experience	15
I have withdrawn from social activities I used to enjoy	14
I am isolating myself from others	14
I feel anxious in social situations	12
I am drained of energy after social interactions	12
I struggle with finding the motivation to see friends or family	12
I feel like a need to hide or ‘mask’ my CRCI from others	11
I feel overwhelmed in social situations	11
I avoid social interactions at work (e.g. at lunchtime)	7
I have withdrawn from established relationships with friends, family, or colleagues	7
I find small talk exhausting	7
I try to find excuses to not see others (e.g. white lies to avoid social interactions)	5
I find myself ‘faking it’ in social situations	5
I want to avoid seeing friends or family	3
I have stopped arranging catch-ups with family and friends	2
I wish to be alone	2
I have a preference for solitary activities	1
I find I get irritated by others	1
**Theme 6 (from steps #1 and #2): Informational needs**	
**Item**	**N Endorsement**
I need to be informed about what things I can do to help myself manage or improve	18
I sometimes fear that I am losing my mind, going crazy, or that I am experiencing early signs of dementia	15
I need help finding or accessing resources (e.g. information sheets, brochures) that I can give to others (friends, family, employers) to help them understand CRCI	14
I need information about what to expect about CRCI (e.g. how long it will last, whether it changes with time)	14
I am not sure if my experience is normal	14
I need help finding resources (e.g. information sheets, brochures) to help me understand CRCI	10
I need help understanding what CRCI is	8
I need to have CRCI explained to me effectively by a health professional	7
I need access to a doctor who understands my experience with CRCI	7

SUPPLEMENTARY MATERIALS

I need help understanding why I am experiencing cognitive difficulties	6
I need help finding/getting in touch with others who experience CRCI (e.g. support groups, groups on social media)	6
I need resources I can show my employer about CRCI to help them understand	6
I need a professional to go over CRCI because I received too much information when my treatment began	5
I need information about how long CRCI will last for	5
I need help to understand how common my experience is	3
I need help finding information about CRCI	2

SUPPLEMENTARY MATERIALS

**Supplementary Table 3. Sample Characteristics for Cancer Survivor Participants Providing Feedback in Round 1**	
**Characteristic**	**Mean (SD)/Range/Count**
**Age (years)**	M = 59.9 years (SD = 5.43, Median = 60 Min = 49, Max = 67)
**Gender**	
Male	1 (10.0%)
Female	9 (90.0%)
**Self-Identified Ethnicity**	
Australian	6 (60.0%)
Anglo-Irish	1 (10.0%)
Maltese/Scottish	1 (10.0%)
Anglo-Celtic	1 (10.0%)
**Primary Cancer Type**	
Breast	6 (60.0%)
Lymphoma	3 (30.0%)
Ovarian	1 (10.0%)
**Employment Status**	
Not currently employed	1 (10.0%)
Part-Time	2 (20.0%)
Full-Time	2 (20.0%)
Casual	2 (20.0%)
Retired	3 (30.0%)
Home Duties	1 (10.0%)
**Education**	
Secondary School	2 (20.0%)
Vocation	5 (50.0%)
Bachelor’s degree	2 (20.0%)
Master’s Degree	1 (10.0%)
**Occupation Sector**	
Healthcare	1 (10.0%)
Management	1 (10.0%)
Education	2 (20.0%)
Creative work	1 (10.0%)
Police force	1 (10.0%)
**When did you first perceive you had ‘brain fog’**	
Before treatment	1 (10.0%)
During treatment	3 (30.0%)
After treatment	6 (60.0%)
**At what stage did you perceive ‘brain fog’ was at its worst?**	
During treatment	1 (10.0%)
After treatment	9 (90.0%)
**Treatments received**	
Chemotherapy	10 (100.0%)
Radiation	5 (50.0%)
Hormone treatment	4 (40.0%)
Surgery	7 (70.0%)
Stem cell transplant	1 (10.0%)
Immunotherapy	
**Time since completion of active treatment**	M = 4.73 years (SD = 5.79, Min = 8 months, Max = 18.8 years)
Note. Ethnicity was self-identified through an open response.	

SUPPLEMENTARY MATERIALS

**Supplementary Table 4. Sample Characteristics for Cancer Survivor Participants Providing Feedback in Round 2**	
**Characteristic**	**Mean (SD)/Range/Count**
**Age (years)**	M = 56.37 years (SD = 8.85, Min = 39, Max = 75)
**Gender**	
Male	5 (26.3%)
Female	14 (73.7%)
**Self-Identified Ethnicity**	
Australian	15 (79.0%)
Anglo-Irish	2 (10.5%)
New Zealand	1 (5.3%)
Anglo-Celtic	1 (5.3%)
**Primary Cancer Type**	
Breast	12 (63.2%)
Lymphoma	5 (26.3%)
Leukemia	1 (5.3%)
Ovarian	1 (5.3%)
**Employment Status**	
Not currently employed	2 (10.5%)
Part-Time	3 (15.8%)
Full-Time	7 (36.8%)
Casual	2 (10.5%)
Retired	5 (26.3%)
**Education**	
Secondary School	4 (21.1%)
Vocation	9 (47.3%)
Bachelor's degree	5 (26.3%)
Master's Degree	1 (5.3%)
**Occupation Sector**	
Administration & Reception	3 (15.8%)
Transportation	1 (5.3%)
Management	2 (10.5%)
Education	2 (10.5%)
Creative work	1 (5.3%)
Data analysis	1 (5.3%)
Social Services	1 (5.3%)
Service professional	1 (5.3%)
Police force	1 (5.3%)
**When did you first perceive you had 'brain fog'**	
Before treatment	1 (5.3%)
During treatment	7 (36.8%)
After treatment	11 (57.9%)
**At what stage did you perceive 'brain fog' was at its worst?**	
During treatment	2 (10.5%)
After treatment	17 (89.5%)
**Treatments received**	
Chemotherapy	12 (63.2%)
Radiation	9 (47.4%)
Hormone treatment	8 (42.1%)
Surgery	10 (52.6%)
Stem cell transplant	3 (15.8%)
**Time since completion of active treatment**	M = 6.19 years (SD = 6.73, Min =1 month, Max = 23 years)
Note. Ethnicity was self-identified through an open response. 7 consumers provided feedback for both round 1 and round 2	

SUPPLEMENTARY MATERIALS

Supplementary Table 5. Sample Characteristics of Cancer Survivors Completing the Validation Survey	
**Characteristic**	**Mean (SD)/Range/Count**
**Age (years)**	M = 44.39 years (SD = 15.02, Min = 18, Max = 81)
**Sex at Birth**	
Male	132 (26.9%)
Female	359 (73.1%)
**Ethnicity**	
Caucasian	290 (59.1%)
African/African American	152 (31.0%)
Asian	17 (3.5%)
Hispanic or Latino	12 (2.4%)
Native American / American Indian	3 (0.6%%)
Other	17 (3.5%)
**Primary Cancer Type**	
Breast	169 (34.4%)
Prostate	22 (4.5%)
Bowel/Colorectal	32 (6.5%)
Melanoma	18 (3.7%)
Lung	26 (5.3%)
Lymphoma	39 (7.9%)
Leukemia	28 (5.7%)
Brain	14 (2.9%)
Pancreatic	3 (0.6%)
Myeloma	3 (0.6%)
Cervical	21 (4.3%)
Thyroid	30 (6.1%)
Testicular	17 (3.5%)
Uterine	14 (2.9%)
Ovarian	19 (3.9%)
Sarcoma	5 (1.0%)
Kidney	5 (1.0%)
Bladder	4 (0.8%)
Other	22 (4.5%)
**Country of Residence**	
USA	175 (35.6%)
Australia	15 (3.1%)
Austria	1 (0.2%)
Belgium	1 (0.2%)
Canada	11 (2.2%)
Czech Republic	1 (0.2%)
Denmark	1 (0.2%)
UK	113 (23.0%)
France	7 (1.4%)
Germany	3 (0.6%)
Greece	2 (0.4%)
Hungry	1 (0.2%)
Ireland	3 (0.6%)
Italy	6 (1.2%)
Kenya	3 (0.6%)
Mexico	5 (1.0%)
New Zeeland	4 (0.8%)
Poland	10 (2.0%)
Portugal	5 (1.0%)
Slovenia	2 (0.4%)
South Africa	119 (24.2%)
Spain	2 (0.4%)
Sweeden	1 (0.2%)
**Employment Status**	
Not currently employed	54 (11.0%)
Part-Time	67 (13.6%)
Full-Time	285 (58.0%)
Casual	6 (1.2%)
Retired	53 (10.8%)
Student	11 (2.2%)

SUPPLEMENTARY MATERIALS

Home Duties	15 (3.1%)
**Highest Level of Education**	1 (10.0%)
Primary/Elementary School	1 (0.2%)
Secondary School	79 (16.1%)
Vocation/Technical School	82 (16.7%)
Bachelor's degree	189 (38.5%)
Master's Degree	110 (22.4%)
PhD/MD	30 (6.1%)
**When did you first perceive you had 'brain fog'**	
Before treatment	77 (15.7%)
During treatment	262 (53.4%)
After treatment	152 (31.0%)
**At what stage did you perceive 'brain fog' was at its worst?**	
Before treatment	40 (8.1%)
During treatment	216 (44.0%)
After treatment	235 (47.9%)
**Severity of Brain Fog Compared to Cognitive Functioning Before Cancer (1-10)**	M = 4.63 (SD = 2.39)
**Severity of Brain Fog Compared to Brain Fog at its Worst (1-10)**	M = 4.36 (SD = 2.52)
**Treatments received**	
Chemotherapy	336 (68.4%)
Radiation	256 (52.1%)
Hormone treatment	133 (27.1%)
Targeted Therapies	66 (13.4%)
Surgery	110 (22.4%)
Immunotherapy	78 (15.9%)
Other	11 (2.2%)
Note. Rounding and multiple endorsements may result in percentages not equalling 100%.	
